# Solid State Characterization of Domperidone: Hydroxypropyl-β-Cyclodextrin Inclusion Complex

**DOI:** 10.4103/0250-474X.65032

**Published:** 2010

**Authors:** D. S. Ghodke, G. M. Chaulang, K. S. Patil, P. D. Nakhat, P. G. Yeole, N. S. Naikwade, C. S. Magdum

**Affiliations:** Institute of Pharmaceutical Education and Research, Borgoan, Meghe, Wardha-442007, India; Appasaheb Birnale College of Pharmacy, South Shivajinagar, Sangli-416416, India

**Keywords:** Domperidone, HP-β-CD, inclusion complex

## Abstract

The purpose of the present study was to prepare inclusion complex of domperidone with hydroxylpropyl-β-cyclodextrin in order improved the solubility and hence to increase dissolution of domperidone. An effect of concentration of hydroxylpropyl-β-cyclodextrin on the aqueous solubility of domperidone was determined by phase-solubility method. The aqueous solubility of domperidone increased as a function of hydroxylpropyl-β-cyclodextrin concentration, showing AL type diagram. Solid domperidone/hydroxylpropyl-β-cyclodextrin complex was prepared in ratio 1:1 by ultrasonication and kneading method. Solid state inclusion complex was characterized by FTIR, powder X-ray diffraction and differential-scanning calorimetry techniques. FTIR studies showed intactness of drug in complex whereas powder diffraction studies showed that hydroxylpropyl-β-cyclodextrin complex was amorphous. Solubility studies showed that complexation increased domperidone solubility as compared to pure drug in 0.1M hydrochloric acid and distilled water. Drug content confirms that ultrasonication is one of the efficient methods to prepare inclusion complex. Dissolution data of inclusion complexes also indicated that there is 1.4 folds increase in dissolution as compared to pure drug and was observed in case of inclusion complexes prepared by ultrasonication.

Domperidone (DPD) is a widely used antiemetic, poorly water soluble drug, erratically absorbed in stomach with several dissolution related problems leading to poor bioavailability[1]. Complexation has been frequently used to increase the aqueous solubility and dissolution rate of water insoluble and slightly soluble drugs in an effort to increase oral bioavailability. However, in certain instances, this approach can also be used to increase drug stability (13-cis-retinoic[2] , disoxaril[3]), control drug release rate (nicardipine[4] , vinpocentine[5] , fentanyl[6] ), improve organoleptic properties and maximize the gastrointestinal tolerance by reducing drug irritation after oral administration. Amongst different β-cyclodextrins (β-CDs), hydroxylpropyl-β-cyclodextrin (HP-β-CD) was selected considering its greater water solubility of ~ 45% w/v over β-cyclodextrin (~ 2% w/v)[7].

In our work, inclusion complexes have been prepared of HP-β-CD and DPD in solid state. Complex formation was confirmed using a variety of analytical techniques as well as dissolution and solubility characteristics. The aim of this work was to evaluate the effect of the different preparation methods on the physicochemical properties of DPD in the binary mixtures. Knowledge of the interaction between the DPD and the cyclodextrin derivative may provide useful information on the development of pharmaceutical forms for oral administration.

Domperidone (IPCA Laboratories, Ratlam, India), and HP-β-CD (Roquette Fereres, France) were received as gift sample. All other chemicals used in the study were of analytical grade and used as received.

Phase solubility studies were performed according to the method reported by Higuchi and Connors[8,9]. An excess amount of DPD was added to 10 ml of distilled water containing increasing concentration HP-β-CD solution (0.01-0.1 M) in 10 ml screw capped bottles. The contents were stirred for 72 h at 37° on a rotary flask shaker. After equilibrium, the samples were filtered through Whatman filter paper No. 42 and absorbances were recorded at 284 nm.

The apparent stability constant was calculated from the initial straight portion of the phase solubility diagram using the Eqn. *K*1:1=slope/So(1-slope) × *M*-1, where, ‘So’ is solubility of drug without cyclodextrin, M is molar concentration, K is apparent stability constant and slope is calculated from regression Eqn.

Inclusion complex was prepared according to method described by Liu, *et al*[8]. DPD and the HP-β-CD (USHP1) were weighed (1:1 molar ratio) and transferred to beaker containing equivolume mixture alcohol-water, sufficient solvent was added to maintained paste like consistency. The resulting paste was then sonicated for 6 h. Throughout the ultrasonication, a paste like consistency was maintained using alcohol-water. Then it was dried in an oven at 50° for 24 h. The dried complexes were passed through sieve No. 100. The prepared complexes were stored in glass vials and used for further studies[10].

DPD and HP-β-CD (KNHP1) were weighed (1:1 molar ratio) and transferred to a mortar and kneaded for 45 min, using equivolume alcohol-water mixture, sufficient solvent was added to maintain a paste like consistency. The resulting paste was then dried in the oven at 50° for 24 h. The dried complexes were ground in a mortar for 2 min and passed through sieve No. 100. The prepared complexes were stored in glass vials and used for further studies[11]. Physical mixtures[12] were prepared by simple blending of DPD and HP-β-CD in a 1:1 molar ratio uniformly in a mortar.

The DSC study was carried out with Mettler DSC 30S, Mettler Toledo India Pvt. Ltd., Switzerland, using crucible Al 40 µl, at of 10° /min heating rate, under nitrogen environment. The temperature range used was 0-400°. Powder XRD was carried out with X-ray powder diffraction system, PANlytical Spectris Pvt. Ltd., Singapore using copper target, a voltage of 40 Kv and a current of 30 mA. The scanning was done over 2θ range of 5° to 60°. The FT-IR spectra of pure drug, pure M-β-CD, physical mixtures and inclusion complex were taken by preparing KBr pellets using pressure 6-8 tons, die size 13mm and scan between 4000-500 cm^−1^.

The percent drug content of each inclusion complex was determined using powder equivalent to 10 mg DPD and was dissolved in 20 ml 0.1M HCl using the mechanical shaker for 20 min. and to the solution obtained 0.1M HCl was added, volume was made up to 50 ml. The solution was then filtered through Whatman filter paper No.42 and required dilutions were being made and absorbance was taken at 284.20 nm.

Inclusion complexes equivalent to 10 mg of DPD was taken and to this 10 ml of the respective medium was being added in 100 ml stoppered volumetric flasks and shaken for 24 h at room temperature (25) on a mechanical shaker. After 24 h, samples were filtered through Whatman filter paper No.42 and aliquots were suitably diluted for estimating solubility[13].

Dissolution studies on pure drug and inclusion complexes (equivalent to 10 mg of drug) were performed using USP II (Rotating paddle type) at 100 rpm. Hydrochloric acid at pH 1.2 (0.1 M 900 ml) maintained at 37±0.5° was used as dissolution media. During dissolution study 10 ml aliquot was withdrawn at different time intervals of 5, 10, 15 up to 60 min. and same was replaced with equal volume of fresh medium. The withdrawn samples were filtered through Whatman filter paper No. 42 and absorbances were measured at 284.20 nm. Cumulative percent drug dissolved was found out at each time interval and graph was plotted between cumulative % drug dissolved and time in min.

The inclusion complex (USM1) was selected as optimized inclusion complex and stability study was carried out at[13] 25±2° at 75±5% RH and 40±2° at 75±5% RH, for a period of 3 months. The inclusion complexes were placed in amber coloured bottles and put at above specified conditions for 3 months. After every month inclusion complexes were analyzed for drug content.

From the phase solubility study it was observed that HP-β-CD showed A_L_ type phase solubility curve indicated improved solubility. Solubility of DPD increased in a linear fashion with increased concentration of HP-β-CD (R^2^ = 0.990) and showed A_L_ type phase solubility curve indicating that soluble complexes were formed and no precipitation was observed (fig. 1). This fact is well supported by Challa *et al*[14]. The stability constant (Ks) of the 1:1 complex of DPD with H-β-CD was calculated from slope of straight line in A_L_ type solubility diagram and was found to be following 87.33 M^−1^. K_S_ values obtained is less but adequate for the formation of inclusion complexes which may contribute improving the bioavailability of poorly water soluble drugs.

**Fig. 1:**
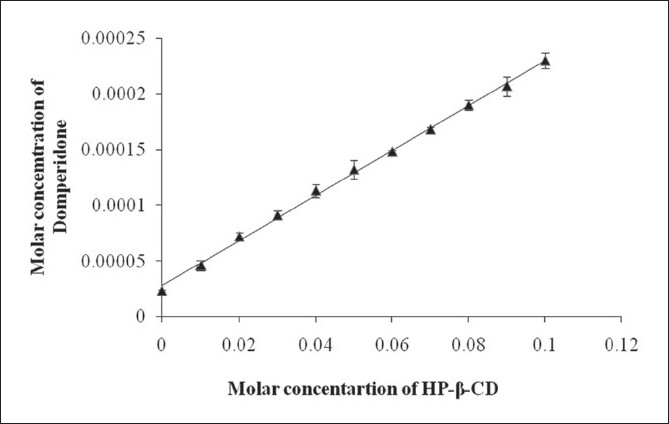
Phase solubility studies of DPD

The FT-IR of pure DPD is characterized by N-H stretching: 3122 cm^−1^ , C=O stretching: 1714.60 cm^−1^ , indicating the presence of -CONH group. Asymmetric C-H stretching: 2937.38 cm^−1^, symmetric C-H stretching: 2817.81 cm^−1^ , N-H deformation: 1693.38 cm^−1^ , aromatic C-H stretching: 3024.18 cm^−1^ , C=C: 1622.02 cm^−1^. The FT-IR of pure HP-β-CD is characterized by OH-stretching: 3442.70 cm^−1^ and 3300.84 cm^−1^. C=O stretching : 1159.14 cm^−1^, C=C stretching : 1633.59 cm^−1^. In all the inclusion complexes the prominent and characteristics peaks of DPD are appeared indicating intactness of drug in complexes (fig. 2).

**Fig. 2:**
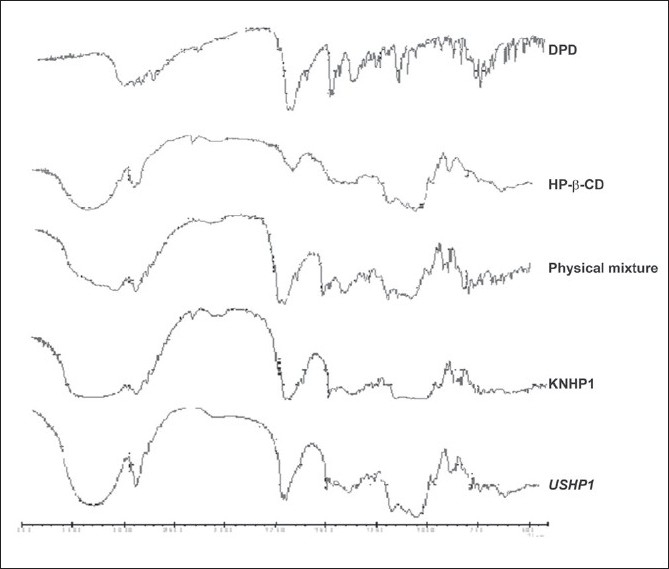
FT-IR Studies of DPD, HP-β-CD, physical mixture and inclusion complexes

The X-Ray diffraction pattern of DPD exhibited sharp, highly intense and less diffused peaks indicating the crystalline nature of drug. The X-Ray diffraction pattern of physical mixture of DPD with HP-β-CD was simply a superimposition of each component with peaks of both DPD and carriers however with lower intensity. The kneaded and sonicated inclusion complexes showed less intense and highly diffused peaks of drug which was very poor in reflections which testified to a reduced ordering of crystal lattice indicating formation of amorphous solid state (fig. 3). The extent of crystallinity influences the dissolution of a drug. An amorphous, less crystalline and metastable form as compared to pure drug dissolves at a faster rate because of high internal energy and greater molecular motion which enhance the thermodynamic property as compared to crystalline materials[14]. In the prepared inclusion complexes there was a reduction in crystallinity of the drug as compared to pure sample which reflects that the drug is dispersed in the polymer and hence increases in the solubility as compared to pure drug. The thermal curve of DPD (T_peak_ =251.6°, ΔH=150.9 J/g_-_ ) indicated its crystalline anhydrous state. Marked reduction of area, broadening and down shifting of peak temperature of drug melting endotherm (T_peak_ =246.7°, Δ H=31.4 J/g), were observed in physical mixture with HP-β-CD, indicative of a more evident loss of drug crystallinity.

**Fig. 3:**
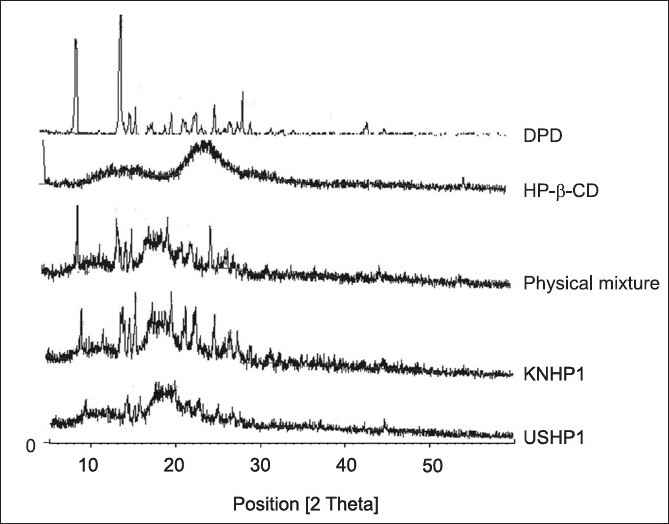
X-ray studies of DPD, HP-β-CD, physical mixture and inclusion complexes

In all the inclusion the drug melting endotherm broaden and shifted to lower temperature passing from physical mixture (T_peak_ =248.7°, ΔH=39.3 J/g) to kneaded 1:1 (242.9°, ΔH=35.5 J/g) to ultrasonication 1:1 (T_peak_ =240.9°, ΔH=14.9 J/g). In all the inclusion complexes, the drug endothermal effect further broadens and was almost hidden by the dehydration band of the carrier and it finally disappeared in the ultrasonication 1:1 (fig. 4). This last phenomenon is attributable to both, inclusion complexes formation and/or drug amorphization Mura *et al*[15].

**Fig. 4:**
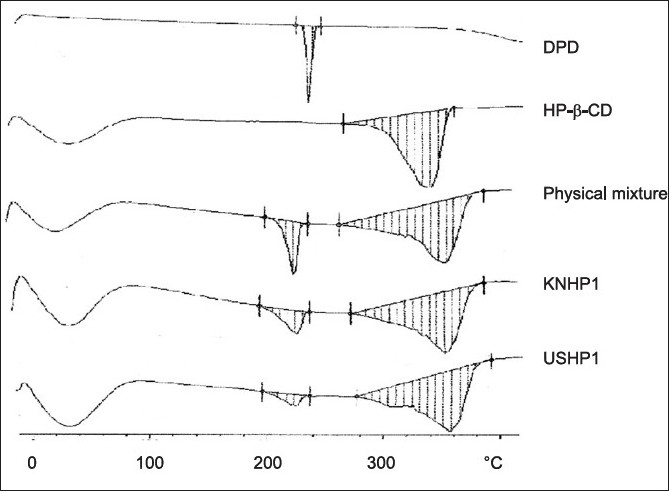
DSC Studies of DPD, HP-β-CD, physical mixture and inclusion complexes

Drug content of all inclusion complexes were in the range of 78.94-88.43%. This indicates the proper loading of drug in inclusion complexes and effectiveness of kneading method and ultrasonication. The solubility of all inclusion complexes was studied in distilled water and 0.1M HCl. The data indicated (Table 1) that solubility increased in all cases but highest increase was found in inclusion complexes prepared by ultrasonication.

**Table 1:** SOLUBILITY STUDIES OF PURE DOMPIDONE, HP- β-CD, PHYSICAL MIXTURE AND INCLUSIONCOMPLEXES

Inclusion complexes	Solubility in distilled water (mg/100ml)	Solubility in 0.1 M HCl (mg/100ml)
DPD	0.4933±0.002	0.72±0.00001
Physical mixture	0.602±0.0002	0.7266±0.0006
KNHP1	1.59±0.098	1.96±0.0001
USHP1	1.879±0.037	2.179±0.0001

The mean dissolution curve of DPD from various binary systems with CD‘s is present in (fig. 5). It is evident at a glance that all system with CD‘s exhibited better dissolution properties than pure drug alone. Statistically significant differences in term of dissolution were found in all the DPD with HP-β-CD reflecting stronger interaction. The greater ability of HP-β-CD in DPD amorphization could explain the better dissolution properties of the drug. As for the influence of the preparation method, the greatest improvement of drug dissolution was obtained with ultrasonication product, followed in order by kneading and finally by physical mixture. The increased dissolution rate (physical mixture) is attributable both to improvement in drug wettability and to formation of readily soluble complexes in dissolution medium. Further improvement obtained with kneading and ultrasonication could be explain by both the more intimate contact between drug and carrier and the decrease of drug crystallinity, as well as a phenomenon of at least partial drug inclusion complexation. On the contrary, the influence of the preparation method was clearly more marked in case of product with HP-β-CD, where kneaded and ultrasonication product showed an increase in dissolution efficiency of 90 or 110%, in comparison to corresponding physical mixture. The best performance of these product seemed to confirm that drug inclusion complexation occurred substantially only in such systems, thus allowing to obtain the highest dissolution improvement.

**Fig. 5:**
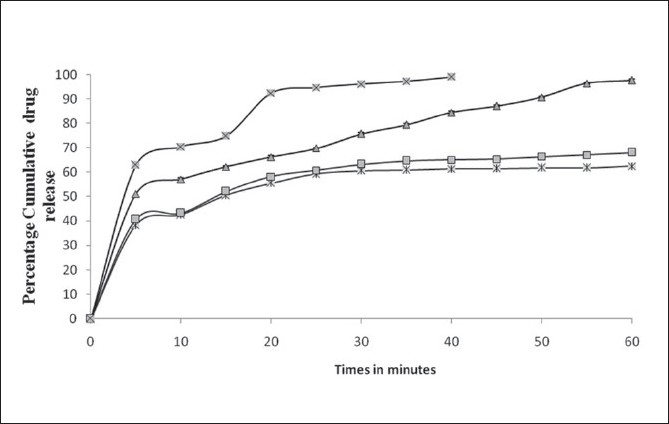
Cumulative % drug release of DPD from physical mixture and inclusion complexes Release of pure domperidone (DPD) alone (–*–) and from physical mixture (–□–), inclusion complex by kneading (–Δ–), and inclusion complex by ultrsonfication (–×–)

Dissolution data of inclusion complexes also indicated that there is increase in dissolution as compared to pure drug and maximum increase was observed in case of inclusion complexes prepared by ultrasonication. Results of stability study indicated that the inclusion complex (USHP1) was stable and there was no significant changes observed in the drug content (*P*>0.05).
